# Combination of aprepitant, palonosetron and dexamethasone as antiemetic prophylaxis in lung cancer patients receiving multiple cycles of cisplatin-based chemotherapy

**DOI:** 10.1111/j.1742-1241.2012.02969.x

**Published:** 2012-08

**Authors:** F Longo, G Mansueto, V Lapadula, L Stumbo, G Del Bene, D Adua, L De Filippis, E Bonizzoni, S Quadrini

**Affiliations:** 1 Medical Oncology A, Policlinico Umberto Primo – SapienzaViale del Policlinico 155, Roma, Italy; 2 Medical Oncology Unit, Fabrizio Spaziani HospitalVia Armando Fabi, Frosinone, Italy; 3 Department of Occupational Health L. Devoto, Section of Medical Statistics and Biometry G. A. Maccacaro, Faculty of Medicine and Surgery, University of MilanMilan, Italy

## Abstract

**Introduction:**

With repeated courses of chemotherapy, chemotherapy-induced nausea and vomiting (CINV) becomes progressively more difficult to control. The aim of this study was to evaluate whether the antiemetic efficacy of the triple combination aprepitant, palonosetron and dexamethasone could be sustained for up to six cycles of highly emetogenic chemotherapy (HEC) (cisplatin ≥ 50 mg/m^2^).

**Methods:**

Chemotherapy-naive patients receiving cisplatin-based HEC, were treated with palonosetron 0.25 mg/i.v., dexamethasone 20 mg/i.v. and aprepitant 125 mg/p.o. 1 h before chemotherapy. Aprepitant 80 mg/p.o. and dexamethasone 4 mg/p.o. were administered on days 2–3. The primary endpoint was complete response (CR, no vomiting and no use of rescue medication), over 5 days following HEC in up to six cycles. Secondary endpoints were emesis-free and nausea-free rates. Safety was also evaluated.

**Results:**

One hundred and fifty six lung cancer patients were included in the study; the median age was 64 years and 76.9% were men. The minimum cisplatin dosage was 75 mg/m^2^, and in most patients was combined with another drug (87.4%). CR ranged from 74.4% (first cycle) to 82% (sixth cycle). More than 90% and 60% of patients were emesis-free and nausea-free during all chemotherapy cycles. The most commonly reported side effects were constipation and headache.

**Conclusions:**

The triple combination of aprepitant, palonosetron and dexamethasone enhanced not only the antiemetic protection during the first cycle, but its efficacy was also sustained for up to six cycles of cisplatin-based HEC in lung cancer patients.

**What's known**

It is already known that palonosetron and aprepitant are efficacious to prevent nausea and vomiting induced in patients receiving high emetogenic chemotherapy. The efficacy of a triple combination palonosetron, aprepitant and dexamethasone to prevent CINV has been assessed only during one chemotherapy cycle (the first).

**What's new**

This article is the first investigating the efficacy of a triple combination palonosetron, aprepitant and dexamethasone in an homogeneous population of lung cancer patients receiving high emetogenic chemotherapy over multiple cycle. The CINV control could help patient to complete all planned chemotherapy cycles. Completion of treatment is essential to give lung cancer patients the maximum chance of treatment success.

## Introduction

Lung cancer is the most common cause of death in oncology patients worldwide (1). Cisplatin-based chemotherapy is the gold standard for treatment in lung cancer patients with locally advanced or metastatic disease (2). Completion of all planned chemotherapy cycles is essential to give patients the maximum chance of treatment success (2,3). Chemotherapy-induced nausea and vomiting (CINV) are among the most distressing symptoms reported by lung cancer patients treated with cisplatin-based chemotherapy (2,3). According to the most recent international guidelines (4,5), cisplatin-based (at dose of ≥ 50 mg/m^2^) regimens are considered to be highly emetogenic forms of chemotherapy (HEC), with more than a 90% risk of inducing CINV (6).

The introduction of first generation 5-HT3 receptor antagonists (RAs), ondansetron, granisetron, dolasetron and tropisetron, into routine oncology practice was a major advance in CINV control, and along with other supportive care led to a major shift in oncology care in the ambulatory setting (7). The second generation 5-HT3-RAs, palonosetron (8–12), the first NK1-RA, aprepitant (13–15) and the use of dexamethasone (16,17) have been shown to further enhance the efficacy of antiemetic prophylaxis. Antiemetic guidelines suggest using a combination of NK1-RA (3 days), 5-HT3-RA (day 1) and dexamethasone (3–4 days) to prevent CINV in patients receiving HEC (4,5). The efficacy of antiemetic prophylaxis is usually evaluated during the first chemotherapy cycle as it is known that pretreated patients are at a higher risk of emesis and anticipatory vomiting in the following cycles (18). Few studies have investigated the efficacy of antiemetic prophylaxis in preventing CINV over multiple cycles of high emetogenic chemotherapy (HEC) (19–21). No previous trials have been published in lung cancer patients receiving single-day HEC and a triple combination of palonosetron plus aprepitant and dexamethasone as antiemetic prophylaxis. In a previous study (22), we investigated the efficacy of a triple combination of aprepitant, palonosetron and dexamethasone in cancer patients receiving HEC, obtaining high control of CINV during the first chemotherapy cycle.

On the basis of these results, we assessed the efficacy of aprepitant, palonosetron and dexamethasone in preventing CINV over subsequent cycles of cisplatin-based chemotherapy in lung cancer patients.

## Materials and methods

From October 2009 until December 2010, a prospective, multicentre observational study was conducted in two oncology departments in Italy.

Chemo-naive adult patients with lung cancer who had an ECOG Performance Status (PS) of 0–2 and were scheduled to receive four-to-six consecutive cisplatin-based chemotherapy cycles were eligible for the study. The cisplatin-based regimen had to contain ≥ 50 mg/m^2^ to be defined as HEC according to the Hesketh classification (23).

Main exclusion criteria were: emesis within 24 h before starting chemotherapy; non-controlled metastasis in the brain; previous radiation to the brain, abdomen or pelvis; and any concomitant medication with antiemetic activity or known to induce the cytochrome P450 enzymes (e.g. phenytoin, carbamazepine).

All eligible patients received aprepitant p.o. 125 mg, palonosetron i.v. 0.25 mg and dexamethasone i.v. 20 mg before starting chemotherapy infusion on day 1; aprepitant p.o. 80 mg and dexamethasone p.o. or i.m. 4 mg were administered on days 2 and 3.

Patients recorded all vomiting episodes, any use of rescue medication (metoclopramide i.m. 10 mg plus dexamethasone i.m. 4 mg in case of vomiting, or metoclopramide p.o. or i.m. 10 mg in case of nausea) and the severity of nausea according to a four-point Likert scale (any, mild, moderate or severe nausea) in a study-specific diary. Patients were asked to report any adverse event over the 5 days (0–120 h) after chemotherapy administration and during all planned cycles.

Study endpoints were measured during the overall phase: from the start of chemotherapy to 120 h post administration (0–120 h). Evaluations were repeated during all chemotherapy cycles.

### Endpoints

The primary endpoint of the study was the proportion of patients who achieved a complete response (CR, defined as no emetic episodes and no use of rescue therapy) during the overall phase of all planned chemotherapy cycles. Secondary endpoints were complete control (CC, defined as no emesis, no rescue therapy and no more than mild nausea), proportion of patients without emetic episodes and proportion of patients with no nausea. Patients returned the diary to the investigator before the start of a new chemotherapy cycle.

Treatment safety was evaluated during the study and all adverse events were recorded and graded according to the common terminology criteria for adverse events (CTCAE) from the National Cancer Institute, version 4.0. (http://ctep.cancer.gov/forms/CTCAEv4.pdf).

### Statistical analysis

Demographic data and patient characteristics were examined and reported as frequencies and percentages, whereas continuous variables were reported as medians and ranges.

A generalised mixed linear model with an identity link function (non-canonical link function) and binomial probability distribution was used to estimate CR, CC and emesis-free rates with an associated two-tailed 95% CI at each therapeutic cycle. The mixed model was parameterised using a full Toeplitz variance-covariance matrix which can be viewed as an autoregressive structure with order equal to the number of repeated measures. A Toeplitz structure for the variance-covariance matrix should represent a suitable choice to take into account correlation across repeated measures (cycles) and to adjust for the potential bias caused by incomplete profiles (< 6 cycles). Incomplete profiles may occur because of the physician's decision to administer fewer than six cycles or, to a lesser extent, because of drop-out events. Computations were performed using the GLIMMIX procedure in SAS version 9.1 (Aloxi, Helsinn Birex Pharmaceutical Ltd, Ireland).

## Results

One hundred and fifty six lung cancer patients were enrolled in the study; most of them were men (76.9%) with stage IV disease (74.3%). Median age was 64 years (range 33–81). All patients were treated with an HEC regimen containing a high cisplatin dose (75–100 mg/m^2^). Patient characteristics are listed in Table 1.

**Table 1:** Patient characteristics

Variable	(*N* = 156)
**Age years**	
Median (range)	64 (33–81)
**Gender % (*n*/*N*)**	
Male	76.9 (120)
Female	23.1 (36)
**Site of tumour % (*n*/*N*)**	
Lung	100 (156)
**Stage % (*n*/*N*)**	
Iib	4.5 (7)
IIIa	9 (14)
IIIb	12.2 (19)
IV	74.3 (116)
**Cisplatin dosage (mg/m^2^) % (*n*/*N*)**	
75	45.5 (71)
80	41 (64)
90	6.4 (10)
100	7.1 (11)
**Regimen**	
Cisplatin combined with 2 drugs	87.4 (152)
Cisplatin only	2.6 (4)
**Chemotherapy cycles completed % (*n*/*N*)**	
Yes	83.6 (146)
6 cycles planned	80.2 (118)
4 cycles planned	18.2 (28)
No*	6.4 (10)

* Five patients attributable to progression and three patients attributable to chemotherapy toxicity.

All patients were planned to receive four to six chemotherapy cycles. The majority completed the planned therapeutic scheme (83.6% of patients); 10 patients withdrew from the study because of disease progression (*n* = 7) or chemotherapy toxicity (*n* = 3).

**Figure 1:**
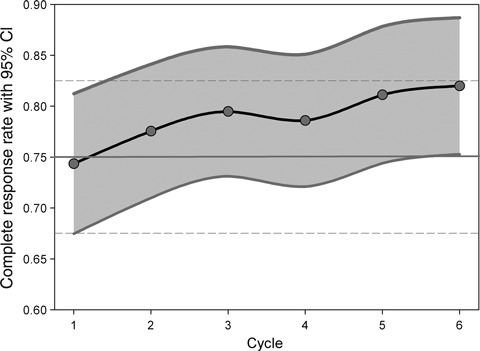
Complete response rate with 95% CI. Complete response is defined as no emesis and no use of rescue medication

The CR, CC and emesis-free rates are reported in Table 2; control of CINV was maintained over all chemotherapy cycles as seen in Figure 1.

**Table 2:** Adjusted rate estimates with two-tailed 95% CIs for patients achieving a complete response, emesis-free and complete control during the overall period (0–120 h after chemotherapy administration) of all chemotherapy cycles

Cycle	*N*	CR Adjusted rate (95% CI)	Emesis-free Adjusted rate (95% CI)	CC Adjusted rate (95% CI)
1	156	0.744 (0.675, 0.812)	0.923 (0.882, 0.964)	0.744 (0.675, 0.812)
2	156	0.776 (0.710, 0.841)	0.929 (0.890, 0.969)	0.776 (0.710, 0.841)
3	156	0.795 (0.731, 0.858)	0.936 (0.898, 0.974)	0.795 (0.731, 0.858)
4	153	0.786 (0.721, 0.851)	0.922 (0.881, 0.964)	0.786 (0.721, 0.851)
5	120	0.811 (0.744, 0.879)	0.906 (0.858, 0.954)	0.811 (0.744, 0.879)
6	118	0.820 (0.753, 0.887)	0.925 (0.881, 0.970)	0.820 (0.753, 0.887)

CI, confidence interval; CR, complete response: no vomiting and no use of rescue medication; emesis-free, no emetic episodes; CC, complete control: no vomiting, no rescue medication and no more than mild nausea.

**Figure 2:**
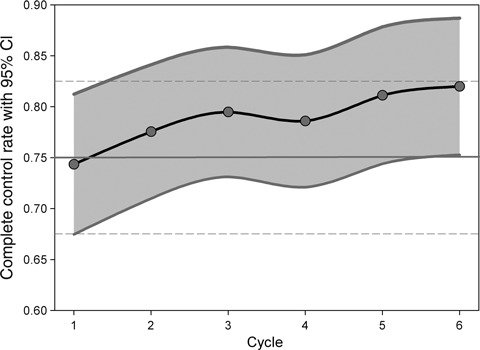
Complete control rate with 95% CI. Complete control is defined as no emesis, no use of rescue medication and no more than mild nausea experience

The maintenance of CC rates (Figure 2) correlated with optimal control of nausea during all subsequent cycles. Severe nausea was not detected during three to six cycles, and nausea was always completely controlled in more than 60% of patients during all completed chemotherapy cycles. Severity of nausea is listed in Table 3.

**Table 3:** Percentage of patients experiencing nausea. Severity of nausea was reported using a four-point Likert scale: no nausea (nausea-free), mild, moderate and severe nausea

Cycle	*N*	No nausea % (*n*)	Mild nausea % (*n*)	Moderate nausea % (*n*)	Severe nausea % (*n*)
1	156	61.5 (96)	30.8 (48)	6.4 (10)	1.3 (2)
2	156	67.4 (105)	22.4 (35)	9.6 (15)	0.6 (1)
3	156	66 (103)	24.4 (38)	9.6 (15)	–
4	153	65.4 (100)	26.1 (40)	8.5 (13)	–
5	120	65.8 (79)	29.2 (35)	5 (6)	–
6	118	70.4 (83)	25.4 (30)	4.2 (5)	–

No grade 3–4 adverse events related to the antiemetic prophylaxis drugs were reported during the study. Grade 1 constipation was reported in 7.1% of patients (11/156), and grade 1 headache in 4.5% of cases (7/156).

## Discussion

The CINV because of cisplatin-based chemotherapy strongly affects the quality of life of cancer patients, and nausea and vomiting still rank among the most distressing symptoms reported (24,25). In lung cancer patients, the completion of all planned chemotherapy cycles is essential to give patients the maximum chance of treatment success (2,3). A clear need exists to obtain higher antiemetic protection, effective during the entire course of chemotherapy with no reduced efficacy (2). Cancer patients receiving HEC are at high risk of CINV (> 90% frequency of emesis), and for this reason antiemetic guidelines suggest using a triple combination of NK1-RA, 5-HT3RA and dexamethasone (4,5). Antiemetic prophylaxis should start at the first chemotherapy cycle, and in the case of successful protection the antiemetic regimen should be repeated over multiple cycles (4,5). A recent trial assessed the efficacy of a triple combination of NK1-RA, 5-HT3RA and dexamethasone as antiemetic prophylaxis in patients receiving cisplatin-based HEC during the first chemotherapy cycle (22). In this trial, aprepitant was used as the NK1-RA, whereas palonosetron was the 5-HT3 RA of choice because of its higher clinical efficacy compared with first generation 5-HT3RAs. On the basis of the results of that trial, we investigated the efficacy of a triple combination of aprepitant, palonosetron and dexamethasone over multiple cycles of cisplatin-based HEC. Few studies have investigated the efficacy of a triple combination in this setting (20,21). De Wit and colleagues reported that the antiemetic efficacy with a first generation 5-HT3 RA was not maintained over repeated highly emetogenic chemotherapy cycles (20). Patients treated with HEC (cisplatin ≥ 70 mg/m^2^) received granisetron as antiemetic prophylaxis (3 mg the day of chemotherapy and 1 mg/bid from days 2 to 7) plus dexamethasone (10 mg the day of chemotherapy and 8 mg/bid from days 2 to 7) (20), and only the addition of aprepitant to ondansetron has been reported to enhance control of CINV in this population (21). In the second trial, De Wit and colleagues randomised patients into two different antiemetic regimens: group A received aprepitant (125 mg on day 1 and 80 mg on days 2 and 3) plus ondansetron (32 mg on day 1) and dexamethasone (20 mg day 1 and 8 mg from days 2 to 5); whereas group B received placebo instead of aprepitant plus ondansetron (32 mg on day 1) and dexamethasone (20 mg on day 1 and 8 mg from days 2 to 5). The CR rates were, respectively, 64% (group A) and 49% (group B) during the first cycle of chemotherapy, and 59% (group A) and 34% (group B) during the sixth cycle. Compared with the antiemetic prophylaxis in group B, patients who received the aprepitant regimen had better and more sustained protection against CINV over multiple cycles (21).

In our trial, we assessed the efficacy of aprepitant, dexamethasone and palonosetron in 156 lung cancer patients receiving single-day HEC (cisplatin ≥ 70 mg/m^2^) over multiple cycles of chemotherapy. The results confirmed that the high efficacy obtained during the first chemotherapy cycle is maintained over the subsequent cycles. The combination of aprepitant plus palonosetron and dexamethasone as antiemetic prophylaxis obtained a CR rate of 74% and 82% during the first and the last chemotherapy cycles respectively.

In our study, most of the lung cancer patients had stage IV disease, and especially for these patients, the completion of all planned courses of chemotherapy is crucial to achieve a treatment response, such as prolonging the time to progression or palliation of symptoms. Control of CINV could contribute to the completion of planned treatment, limiting the detrimental effects of nausea and vomiting and providing an acceptable quality of life. In conclusion, we observed a high proportion of patients who completed all planned cycles (83.6%), which was likely because of optimal control of CINV (CR rates ranged from 74% to 82%).

The antiemetic prophylaxis of aprepitant plus palonosetron and dexamethasone enhanced not only single-cycle antiemetic protection but it is also maintained over multiple cycles.

## References

[1] Jemal A, Siegel R, Ward E (2009). Cancer Statistic, 2009. CA Cancer J Clin.

[2] Dong X, Huang J, Cao R (2010). Palonosetron for prevention of acute and delayed nausea and vomiting in non-small-cell lung carcinoma patients. Med Oncol.

[3] Feinberg B, Gilmore J, Haislip S (2012). Impact of initiating antiemetic prophylaxis with palonosetron versus ondansetron on risk of uncontrolled chemotherapy-induced nausea and vomiting in patients with lung cancer receiving multi-day chemotherapy. Sup Care Cancer.

[4] Roila F, Herrstedt J, Aapro M (2010). Guideline update for MASCC and ESMO in the prevention of chemotherapy- and radiotherapy-induced nausea and vomiting: results of the Perugia consensus conference. Ann Oncol.

[5] Basch E, Prestrud AA, Hesketh PJ (2011). Antiemetics: American Society of Clinical Oncology clinical practice guideline update. J Clin Oncol.

[6] Hesketh PJ (1999). Defining the emetogenicity of cancer chemotherapy regimens: relevance to clinical practice. Oncologist.

[7] Rubenstein EB (1994). Costs and benefits of outpatient therapy. Sup Care Cancer.

[8] Eisenberg P, Figuero-Vadillo J, Zamora R (2003). 99-04 Palonosetron Study Group. Improved prevention of moderately emetogenic chemotherapy induced nausea and vomiting with palonosetron, a pharmacologically novel 5HT3 receptor antagonist. Results of a phase III, single dose trial versus dolasetron. Cancer.

[9] Gralla R, Lichinitser M, Van Der Vegt S (2003). Palonosetron improves prevention of chemotherapy-induced nausea and vomiting following moderately emetogenic chemotherapy: results of a double-blind randomized phase III trial comparing single doses of palonosetron with ondansetron. Ann Oncol.

[10] Aapro MS, Grunberg SM, Manikhas GM (2006). A phase III, double-blind, randomized trial of palonosetron compared with ondansetron in preventing chemotherapy-induced nausea and vomiting following highly emetogenic chemotherapy. Ann Oncol.

[11] Rubenstein EB, Gralla RJ, Eisenberg P (2003). Palonosetron (PALO) compared with ondansetron (OND) or dolasetron (DOL) for prevention of acute and delayed chemotherapy-induced nausea and vomiting (CINV). Combined results of two Phase III trials. Proc Am Soc Clin Oncol.

[12] Saito M, Aogi K, Sekine I (2009). Palonosetron plus dexamethasone versus granisetron plus dexamethasone for prevention of nausea and vomiting during chemotherapy: a double-blind, double-dummy, randomised, comparative phase III trial. Lancet Oncol.

[13] Hesketh PJ, Grunberg SM, Gralla RJ (2003). The oral neurokinin-1 antagonist aprepitant for the prevention of chemotherapy-induced nausea and vomiting: a multinational, randomised, double-blind, placebo-controlled trial in patients receiving high-dose cisplatin: the aprepitant protocol 052 study group. J Clin Oncol.

[14] Poli-Bigelli S, Rodrigues-Pereira J, Carides AD (2003). Addition of the neurokinin 1 receptor anatagonist aprepitant to standard antiemetic therapy improves control of chemotherapy-induced nausea and vomiting: results from a randomized, double-blind, placebo-controlled trial in Latin America. Cancer.

[15] Schmoll HJ, Aapro MS, Poli-Bigelli S (2006). Comparison of an aprepitant regimen with a multiple-day ondansetron regimen, both with dexamethasone, for antiemetic efficacy in high-dose cisplatin treatment. Ann Oncol.

[16] Italian Group for Antiemetic Research (1998). Double-blind, dose-finding study of four intravenous doses of dexamethasone in the prevention of cisplatin-induced acute emesis. J Clin Oncol.

[17] Grunberg SM (2007). Antiemetic activity of corticosteroids in patients receiving cancer chemotherapy: dosing, efficacy, and tolerability analysis. Ann Oncol.

[18] Grunberg S, Clark-Snow RA, Koeller J (2010). Chemotherapy-induced nausea and vomiting: contemporary approaches to optimal management: Proceedings from a symposium at the 2008 Multinational Association of Supportive Care in Cancer (MASCC) Annual Meeting. Sup Care Cancer.

[19] Schwartzberg L, Jackson J, Jain G (2011). Impact of 5-HT(3) RA selection within triple antiemetic regimens on uncontrolled highly emetogenic chemotherapy-induced nausea/vomiting. Expert Rev Pharmacoecon Outcomes Res.

[20] De Wit R, Van Den Berg H, Burghouts J (1998). Initial high anti-emetic efficacy of granisetron with dexamethasone is not maintained over repeated cycles. Br J Cancer.

[21] De Wit R, Herrstedt J, Rapoport B (2003). Addition of the oral NK 1 antagonist aprepitant to standard antiemetics provides protection against nausea and vomiting during multiple cycles of cisplatin-based chemotherapy. J Clin Oncol.

[22] Longo F, Mansueto G, Lapadula V (2011). Palonosetron plus 3-day aprepitant and dexamethasone to prevent nausea and vomiting in patients receiving highly emetogenic chemotherapy. Sup Care Cancer.

[23] Hesketh PJ, Kris MG, Grunberg SM (1997). Proposal for classifying the acute emetogenicity of cancer chemotherapy. J Clin Oncol.

[24] Lindley C, McCune JS, Thomason TE (1999). Perception of chemotherapy side effects cancer versus non cancer patients. Cancer Pract.

[25] Basch E (2010). The Missing Voice of Patients in Drug-Safety Reporting. N Engl J Med.

